# Changes in frailty conditions and phenotype components in elderly after hospitalization^1^

**DOI:** 10.1590/1518-8345.1417.2905

**Published:** 2017-07-10

**Authors:** Gianna Fiori Marchiori, Darlene Mara dos Santos Tavares

**Affiliations:** 2 Doctoral student, Universidade Federal do Triângulo Mineiro, Uberaba, MG, Brazil.; 3 PhD, Associate Professor, Instituto de Ciências da Saúde, Universidade Federal do Triângulo Mineiro, Uberaba, MG, Brazil.

**Keywords:** Frail Elderly, Longitudinal Studies, Geriatric Nursing

## Abstract

**Objective:**

describing the changes in frailty conditions over the period of a year after hospital discharge, verifying predictive variables for changes in frailty conditions and frailty phenotype components according to worsening, improving and stable groups.

**Method:**

a longitudinal survey carried out with 129 elderly. A structured form for socioeconomic and health data, scales (Geriatric Depression Scale - short form, Katz scale, Lawton and Brody scale) and frailty phenotype according to Fried were used. Descriptive analysis and multinomial logistic regression model (p<0.05) were performed.

**Results:**

we found that 56.7% of older adults changed their condition from non-frail to pre-frail, with no changes from non-frail to frail. Deaths were found between frail and pre-frail elderly. In the worsening group, the increase in the number of morbidities was a predictor for exhaustion and/or fatigue, while in the improving group, increased dependence on instrumental activities of daily living was a predictor for weight loss, and reduced scores indicative of depression due to low level of physical activity.

**Conclusion:**

a greater percentage of changes from non-frail condition to pre-frail older adults were observed, and health variables were only predictive for frailty phenotype components.

## Introduction

The frailty syndrome can be defined as a “biological syndrome with multiple causes, characterized by decreased strength, muscular endurance and physiological function, which results in increased vulnerability of the individual to develop functional dependence and/or death”^1^. It is worth highlighting the existence of several definitions and evaluation methods of this syndrome in the literature^1^. Operationalization, proposed by Fried et al. (2001), resulted in the development of the frailty phenotype, which has predictive validity and has been widely used in population-based studies due to its ability to identify changes between health conditions, constituting a clinical and screening tool^2^. It consists of five components: low walking speed, decreased muscle strength, self-reported exhaustion and/or fatigue, unintentional weight loss and low physical activity level^2^. Impairment present in three or more of these components characterizes a frail older adult; while impairment in one or two components characterizes as pre-frail, and older adults are considered as robust or non-frail in the absence of impairment^2^.

Frailty is considered a dynamic and bidirectional process^3^, characterized by frequent changes in the conditions of frail, pre-frail and non-frail^3^. In this context, it is possible to observe improvement, stability or worsening of the initial condition presented by the older adult^1^ ^,^ ^3^. However, the description of these changes and reasons for their occurrence are still scarce in the national and international literature^4^ ^-^ ^5^.

Frailty is associated with adverse health outcomes such as: morbidities, depression symptoms, decreased functional capacity, institutionalization, hospitalization and mortality^2^. However, further studies on this subject to increase knowledge of the role of these variables in improvement and/or worsening of the frailty condition and its components are still necessary since they result in a greater possibility of recovery by older adults^6^.

Hospitalization, the initial environment of this study, is characterized as a risk factor for development of frailty syndrome^2^, having a negative influence on the change between its conditions after discharge and exposing older adults to the risks of adverse outcomes^5^. Morbidities that result in hospitalizations reduce the chances of improving the frailty condition, and after discharge, older adults with intermittent episodes of new hospitalizations are more likely to have limitations in functional capacity and die^5^. This demonstrates the need for following-up older adults after their discharge, as well as understanding changes in frailty conditions. National studies addressing hospitalized older adults and frailty have been performed with cross-sectional designs^7^ ^-^ ^8^, while changes in frailty conditions have been studied through research with older adults in the community at national^4^ ^,^ ^9^ and international^3^ ^,^ ^5^ ^-^ ^6^ levels. Investigations after hospital discharge were not found.

Among studies with a longitudinal design that have addressed changes in frailty conditions, the frailty phenotype proposed by Fried^4^ ^,^ ^9^ or adaptations of this^3^ ^,^ ^5^ ^-^ ^6^ were used, where the age of the older adults included and the follow-up period both varied between the studies^5^ ^-^ ^6^ ^,^ ^9^. The highest percentage of changes occurred from non-frail to pre-frail older adults^3^ ^,^ ^5^ ^,^ ^9^, while changes from frail to non-frail older adults had the lowest percentage in international^3^ ^,^ ^5^ and national^4^ ^,^ ^9^ studies. An outcome of death was commonly associated with frailty and pre-frailty^3^ ^,^ ^5^ ^-^ ^6^ ^,^ ^9^.

Identifying changes in frailty conditions and their predictive variables allows for evaluating between these events and to estimate the probability of improvement, stability, worsening or death of an individual^6^. It is also capable of assisting health professionals to develop early interventions^6^ ^)^ by guiding care toward health promotion, disease prevention and to control of risk factors that anticipate the onset of frailty syndrome^7^.

This study aimed to describe changes in frailty conditions in the following year after hospital discharge, as well as to verify the predictive variables for changes in frailty conditions and frailty phenotype components according to the following groups: worsening, improving and stable.

## Methods

This is a longitudinal and analytical study, conducted from April 2013 to March 2014, in the inpatient Medical Clinic (MC) and Surgical Clinic (SC) of a university hospital in the interior of Minas Gerais, MG, Brazil, at the time of hospitalization of older adults; and from April 2014 to March 2015, after one year of hospital discharge, in their home. The high complexity university hospital serves 27 municipalities in the macro-region of the South Triangle of Minas Gerais, with 302 beds, 37 for the MC and 65 for the SC.

In order to calculate sample size, a frailty prevalence of 30.0% was considered based on studies with older adults in hospital settings (33.2%^10^ and 37%^11^), thus reaching a sample of 265 elderly with 5% accuracy and 95% confidence interval, for a finite population of 1,455 eligible elderly. The recruitment process was carried out by systematic random sampling with an interval of k=2.

Inclusion criteria considered were: being 60 years of age or more, being hospitalized in the MC and SC wards during the aforementioned period, not having cognitive decline, being able to walk (use mobility aid devices were allowed) and participating in the two stages of the research. As exclusion criterion, the following were considered upon hospital admission and after one year: presenting severe Cerebral Vascular Accident (stroke/CVA) sequelae, with localized loss of strength and aphasia, severe or unstable Parkinson’s disease associated with severe motor, speech, or affective impairment that would make evaluations impossible; in terminal stage, presenting severe vision and hearing deficit, being hospitalized again, having already been interviewed during the collection period or having restriction to move around and talk.

At the hospital, 265 older adults were interviewed; the second collection was performed one year after discharge, and started with 163 older adults, as the others were excluded because they did not complete anthropometric data (10) and the frailty phenotype components (5), and they did not reside in the urban area of the municipality (87). Of the 163 older adults, those who presented stroke sequelae (1) and cognitive decline (1) were excluded, in addition to losses due to refusals (3), death (20), not found after three visits (6), moved to another city (1), were hospitalized (1) and impossibility of carrying out the evaluation (1). Therefore, 129 older adults participated in the second stage of the study.

Data were collected by previously trained interviewers in both study stages. Cognitive screening was done before the interview using the Mini Mental State Examination (MMSE), using the version translated and validated in Brazil, which considers the level of schooling at the cut-off points for cognitive deficit^12^. If the older adult presented cognitive decline in this evaluation, the accompanying person was asked to take part in the PfefferQuestionnaire^13^. If the result was below six, the interview was carried out with the older adult and information supplemented, if necessary, by the companion; if the result was equal to or greater than six, the interview was closed.

A structured form designed by the Research Group on Public Health of the Federal University of the Triângulo Mineiro (UFTM), based on the literature and the researchers’ expertise was applied for socio-demographic, economic and health data characterization. Information on morbidities and the regular use of medications were obtained through reports of the older adults, as well as new hospitalizations. Depression diagnosis was measured using the Geriatric Depression Scale - Short Form (GDS-15), considering a score higher than five as cut-off point^14^. Functional disability was assessed by the Katz Scale^15^ through Basic Activities of Daily Living (BADL) and the Lawton and Brody Scale^16^ ^)^ by Instrumental Activities of Daily Living (IADL).

The frailty syndrome was evaluated through the five phenotype components: 1) unintentional weight loss: evaluated by the following question: “In the last year, have you unintentionally lost more than 4.5 kg (meaning, without dieting or exercising)?”; 2) decreased muscle strength: verified based on palmar grip strength using a SAEHAN® manual hydraulic dynamometer, in accordance with the *American Society of Hand Therapists* recommendations*,* with three measurements and considering their average value - the cut-off points proposed by Fried et al.^2^ were adopted; 3) self-reported exhaustion and/or fatigue: evaluated by two questions of the Brazilian version of the Depression scale *Center for Epidemiological Studies* ^17^, items 7 and 20, where older adults with impairment were considered those with a score of two or three in any of the questions; 4) low walking speed: evaluated by the walking time spent to cover a distance of 4.6 meters, using a professional chronometer as standard - three measurements were taken, using the mean value of these measures and adopting the cut-off points proposed by Fried et al.^2^; and 5) low physical activity level: measured by the weekly energy expenditure in Kcal, measured through the long version of the International Physical Activity Questionnaires (IPAQ), adapted for older adults by Benedetti et al.^18^. Those who spent 150 minutes or more of weekly physical activity were considered active, and those ranging from zero to 149 minutes as inactive. The impairment in 3 or more components of the phenotype classified older adults as frail; between 1 and 2 components as pre-frail, and none as non-frail^2^.

Older adults were categorized according to groups of: Improving (older adults who changed their condition from frail to pre-frail or non-frail, in addition to from pre-frail to non-frail); stable (older adults who maintained the initial condition at the second moment) and worsening (older adults in the non-frail to pre-frail and frail conditions, and from pre-frail to frail).

The study variables were the frailty conditions and frailty phenotype components (improving, stable and worsening groups); outcome of death; difference (dif) between the following values one year after discharge from hospital: a score indicative of depression dif; number of medications dif; number of morbidities dif; BADL score dif; IADL score dif., all were used quantitatively.

An electronic database was built in the Excel® program, the collected data were processed in microcomputer by two people using double entry, where consistency between the fields were verified and inconsistencies were corrected according to the original interview. The database was subsequently imported into the *Statistical Package for Social Sciences* (SPSS) version 20.0 software for descriptive (absolute and percentage frequencies) and multivariable analysis, with estimates of prevalence odds ratios, using the multinomial logistic regression model, considering a level of significance of 5% (p<0.05) and 95% Confidence Interval (CI) for the frailty condition and the frailty phenotype components.

The study was approved by the Committee of Ethics in Research with Human Beings of *UFTM* (Protocol number 2511), and obtained consent from the MC and the SC departments and the Teaching and Research Department of the university hospital. The study participants signed the Free and Informed Consent Form (TCLE) and received a copy at both stages.

## Results

Among the 129 older adults who completed the follow-up, we found higher percentages of frail older adults among females at both moments; and pre-frail and non-frail among males. In the three frailty conditions, the highest percentages in both moments were for older adults between 60 ├ 70 years of age, living with a partner, with 1 ┤4 years of schooling, income of up to 1 minimum wage, without the presence of depression indication and dependents for IADL. At hospital admission, the majority of frail, pre-frail and non-frail older adults were using five or more medications and were independent for BADL. After one year, only pre-frail individuals changed the number of medications used from 1 ┤ 4. A higher percentage of frail older adults with five or more morbidities and who were dependent for BADL was identified after hospital discharge. Pre-frail and non-frail remained with a higher percentage of independence for BADL; however, an increase in the number of morbidities was observed among pre-frail older adults after one year of discharge, while non-frail remained with 1 ┤ 4 (morbidities).

**Figure 1:**
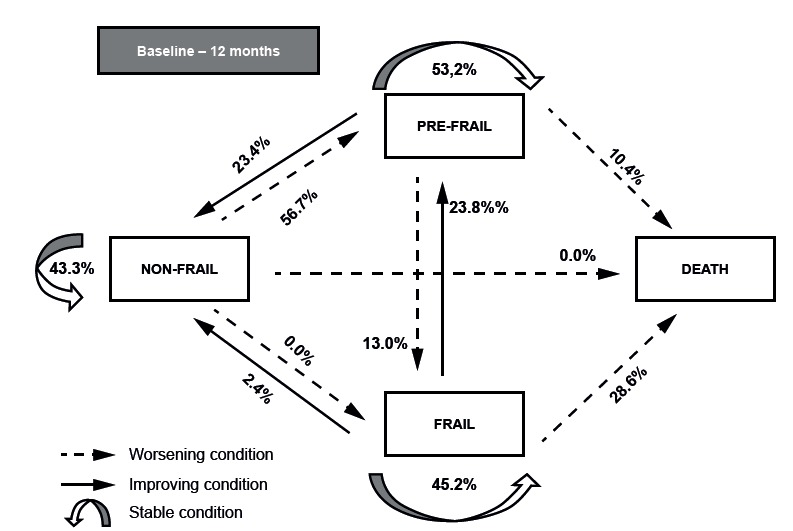
Changes between frailty conditions and death, from baseline to follow-upone year after hospital discharge. Uberaba, MG, Brazil, 2015

In order to analyze the changes in frailty conditions, older adults who participated in both moments (n=129) and those who died during follow-up (n=20) were considered, totaling 149 older adults. The highest percentage was verified for non-frail older adults who had their condition worsened to pre-frail (56.7%), followed by improvement from frail to pre-frail (23.8%). Changes from frail to non-frail represented 2.4%, whereas no changes from the condition of non-frailty to frailty (0%) were observed. The outcome of death had a higher percentage among frail (28.6%) and pre-frail (10.4%) older adults. 45.2% remained stable in the frail condition, 53.2% in the pre-frail and 43.3% as non-frail (Figure 1).

In the final multinomial logistic regression model, we found that variables indicative of depression dif, number of medications dif, number of morbidities dif, BADL score dif and IADL dif were not predictive for changes in the frailty status in the improving and worsening groups (Table 1).

**Table 1:** Final multinomial logistic regression model for the variables indicative of depression, number of medications, number of morbidities, BADL and IADL scores between groups of worsening and improving frailty condition. Uberaba, MG, Brazil, 2015

**Variables**	**Frailty condition**						
	**Improving group**				**Worsening group**		
	OR*	**IC** ^**†**^ **95%**	**p** ^**ǂ**^		OR*	**IC** ^**†**^ **95%**	**p** ^**ǂ**^
**ID score** ^**§**^ **dif**	**0.88**	**0.74-1.05**	**0.155**		**1.00**	**0.84-1.20**	**0.965**
**Number of medications dif**	**0.96**	**0.82-1.13**	**0.649**		**1.06**	**0.90-1.24**	**0.486**
**Number of morbidities dif**	**1.02**	**0.87-1.19**	**0.808**		**1.06**	**0.91-1.23**	**0.478**
**BADL score** ^**||**^ **dif**	**1.26**	**0.79-2.02**	**0.332**		**1.76**	**0.93-3.32**	**0.082**
**IADL score** ^**¶**^ **dif**	**1.13**	**0.81-1.59**	**0.463**		**0.89**	**0.63-1.27**	**0.521**

*OR: *Odds Ratio*; †CI: Confidence interval; ‡p<0.05; §ID: Indicative of Depression; ||BADL: Basic Activities of Daily Living; ¶IADL: Instrumental Activities of Daily Living.

Regarding the frailty phenotype components, the increase in number of morbidities after one year of hospital discharge was 1.26 times more likely to worsen self-reported exhaustion and/or fatigue. In the improving group, the increase in dependence for IADL represented 1.42 times more chance of unintentionally losing weight. In the improving group we found that the reduction of scores indicative of depression was considered a protection factor for low physical activity level (Table 2). No predictors of improving or worsening of the phenotype components of low walking speed and decreased muscle strength were identified (Table 2).

**Table 2:** Final multinomial logistic regression model for variables indicative of depression, number of medications, number of morbidities, BADL and IADL scores, between the worsening and improvinggroups of the frailty phenotype components.Uberaba, MG, Brazil, 2015

**Variables and frailty** **phenotype components** OR*		**Improving group**				**Worsening group**		
		**95%CI** ^**†**^	**p** ^**ǂ**^			OR*	**95%CI** ^**†**^	**p** ^**ǂ**^
**Low walking speed**								
	**ID score** ^**§**^ **dif**	**1.10**	**0.88-1.38**	**0.389**		**0.94**	**0.78-1.14**	**0.553**
	**Number of medications dif**	**1.01**	**0.81-1.26**	**0.939**		**1.15**	**0.97-1.36**	**0.096**
	**Number of morbidities dif**	**0.98**	**0.80-1.19**	**0.837**		**1.09**	**0.92-1.28**	**0.324**
	**BADL score** ^**ǀǀ**^ **dif**	**0.68**	**0.42-1.09**	**0.108**		**1.10**	**0.61-1.99**	**0.753**
	**IADL score** ^**¶**^ **dif**	**1.21**	**0.79-1.83**	**0.379**		**0.97**	**0.67-1.41**	**0.885**
**Decreased muscle strength**								
	**ID score** ^**§**^ **dif**	**1.17**	**0.95-1.45**	**0.145**		**0.89**	**0.64-1.24**	**0.493**
	**Number of medications dif**	**0.86**	**0.69-1.06**	**0.154**		**1.01**	**0.76-1.35**	**0.940**
	**Number of morbidities dif**	**0.98**	**0.81-1.17**	**0.807**		**1.05**	**0.79-1.39**	**0.748**
	**BADL score** ^**ǀǀ**^ **dif**	**0.86**	**0.52-1.42**	**0.556**		**1.21**	**0.47-3.12**	**0.689**
	**IADL score** ^**¶**^ **dif**	**1.09**	**0.74-1.60**	**0.665**		**1.20**	**0.63-2.26**	**0.577**
**Self-reported exhaustion and/or fatigue**								
	**ID score** ^**§**^ **dif**	**0.89**	**0.74-1.08**	**0.244**		**0.81**	**0.62-1.05**	**0.114**
	**Number of medications dif**	**0.93**	**0.76-1.13**	**0.457**		**1.16**	**0.94-1.42**	**0.158**
	**Number of morbidities dif**	**0.84**	**0.70-1.02**	**0.077**		**1.26**	**1.01-1.56**	0.040*
	**BADL score** ^**ǀǀ**^ **dif**	**0.87**	**0.54-1.39**	**0.559**		**0.94**	**0.54-1.63**	**0.830**
	**IADL score** ^**¶**^ **dif**	**0.83**	**0.54-1.26**	**0.379**		**1.29**	**0.82-2.03**	**0.268**
**Unintentional weight loss**								
	**ID score** ^**§**^ **dif**	**0.97**	**0.82-1.15**	**0.725**		**0.98**	**0.76-1.27**	**0.889**
	**Number of medications dif**	**0.92**	**0.78-1.08**	**0.304**		**1.02**	**0.79-1.32**	**0.877**
	**Number of morbidities dif**	**0.97**	**0.83-1.12**	**0.658**		**1.16**	**0.92-1.45**	**0.202**
	**BADL score** ^**ǀǀ**^ **dif**	**1.00**	**0.66-1.52**	**0.981**		**0.44**	**0.18-1.08**	**0.073**
	**IADL score** ^**¶**^ **dif**	**1.42**	**1.02-1.97**	0.038*		**1.12**	**0.63-1.98**	**0.699**
**Low physical activity level**								
	**ID score** ^**§**^ **dif**	**0.75**	**0.58-0.97**	0.030*		**1.06**	**0.88-1.27**	**0.530**
	**Number of medications dif**	**0.87**	**0.69-1.10**	**0.244**		**0.98**	**0.83-1.17**	**0.848**
	**Number of morbidities dif**	**1.05**	**0.84-1.32**	**0.663**		**0.90**	**0.77-1.05**	**0.194**
	**BADL score** ^**ǀǀ**^ **dif**	**1.36**	**0.68-2.72**	**0.377**		**1.24**	**0.72-2.14**	**0.441**
	**IADL score** ^**¶**^ **dif**	**1.24**	**0.75-2.03**	**0.399**		**1.09**	**0.77-1.55**	**0.607**

OR: *Odds Ratio*; †CI: Confidence Interval; ǂp<0.05; §ID: Indicative of Depression; ǀǀBADL: Basic Activities of Daily Living; ¶IADL: Instrumental Activities of Daily Living.

## Discussion

The predominance of older adults who had their frailty condition worsened during follow-up (going from non-frail to pre-frail) is consistent with national studies^4^ ^,^ ^9^ that used Fried’s frailty phenotype. However, they were carried out in the community among older adults aged 65 years and over and for a follow-up period of thirteen^4^ and twelve months^9^. A lower percentage was verified in an international study with older adults of the community aged 70 years or older, using the adapted Fried’s phenotype and a three-year follow-up^5^. Despite the different contexts, the results express the need to develop strategies directed to older adults, especially those in the pre-frail condition, since this condition presents greater sensitivity to interventions, and thus a greater possibility of improving the frailty condition^6^.

Similar percentages were found for changes from frailty to pre-frailty condition in one national study (23.3%)^9^ and one international study (23.0%)^3^, and a lower percentage was found in one other national investigation (7.0%)^4^. Considering the greater vulnerability of older adults hospitalized for developing frailty and negative changes of their conditions^8^, these findings indicate the need for greater follow-up after hospital discharge so that health actions can be implemented early, delaying the onset of frailty.

The findings from a study conducted in Belo Horizonte, MG (2.3%)^9^ corroborate the percentages obtained in the present study regarding changes from frailty to non-frailty conditions. However, a lower percentage was found in another study in Belo Horizonte, MG (0.5%)^4^ and in the United States (0%)^3^, demonstrating the vulnerability of frail older adults and their difficulty in recovering, given their susceptibility to adverse outcomes^2^, especially when hospitalized^8^. An absence of changes from non-frail to frail conditions was also found in Belo Horizonte, MG^4^ ^,^ ^9^, diverging from investigations in the United States^3^ ^,^ ^5^ and in China^6^. The highest percentage of deaths among frail and pre-frail older adults is consistent with national^9^ and international studies^3^ ^,^ ^5^ ^-^ ^6^, emphasizing the association between frailty and mortality^2^.

Among the justifications for the difference in the percentages of changes in frailty conditions, we have: the hospital environment at the start of the follow-up, the characteristics of the populations studied, socioeconomic development, phenotype adaptations and shorter follow-up time when compared to international studies^3^ ^,^ ^5^ ^-^ ^6^. Thus, understanding these changes will allow for developing individualized interventions for different scenarios^8^ ^)^ and investment in care will enable improving health status and/or frailty conditions^6^. From this, a comprehensive evaluation of older adults must be performed by a multiprofessional team, in order to plan the care and interventions able to anticipate development of the frailty syndrome or improve its condition.

Unlike this study, a longitudinal study conducted with older adults in the community of Belo Horizonte, MG, verified the following predictors for worsening of frailty condition in twelve months: history of cancer (OR: 3.4; 95% CI: 1.1-10.9), urinary incontinence (OR: 2.9; 95% CI: 1.3-6.1) and advanced activities of daily living (OR: 1/0.8; 95% CI: 0.6-0.9)^9^.

Corroborating the results of this research, a longitudinal study conducted in Denmark^19^ and a cross-sectional study conducted in a community in Belo Horizonte, MG^20^, found an association between a higher number of morbidities and increased perception of fatigue. The increase in number of morbidities results in a frequent need for therapeutic interventions that indirectly contribute to developing frailty through worsening of its components^21^. In this context, older adults with morbidities have low energy reserves, causing a loss of efficiency and greater energy expenditure doing daily activities, such as decreased walking speed, and thereby worsening exhaustion and/or fatigue^21^. Interventions should be based on improving energy efficiency through compensatory activities such as physical endurance exercises^21^.

The simultaneity of occurrence between self-reported exhaustion and/or fatigue and morbidities^19^ ^-^ ^20^ demonstrates the importance of tracking aspects that characterize such conditions in older adults, the collection of information with this age group about knowledge of their disease and treatment, clarification of doubts and follow-up with the Family Health Strategy, so that there is no development of other diseases.

A cross-sectional study conducted in an outpatient clinic in Turkey with older adults found an association between increased dependence on IADL and worsening of nutritional status, leading to weight loss^22^. Meal preparation activity can be considered an essential factor for malnutrition in older adults and consequently weight loss^22^. When elderly depend on another person to prepare their meals, either because of the environment in which they are inserted, for example in the hospital^23^, or due to the death of their partner who was responsible for performing that task, such condition may be aggravated^23^.

Assessing unintentional weight loss in older hospitalized adults is essential for early and effective interventions^22^. To understand this, the role of IADLs in compromising this component helps to develop strategies directed to specific activities that present limitations among individuals at greater risk. Considered from a comprehensive perspective, the nurse has the accompanying responsibility of older adults through multidimensional evaluation, and ensuring adequate and effective assistance.

The relationship between physical activity and depression is characterized by a bidirectional pathway and considered a relevant topic for health care of older adults^24^. This is corroborated in longitudinal research with older adults in Miami, Florida, where the relationship between increased depression symptoms and decreased physical activity was verified^25^. Diverging from this study, a longitudinal investigation with older adults (*Survey of Health, Aging, and Retirement*) found no significant association between depression symptoms at baseline and increased or decreased level of physical activity at follow-up^24^.

Reduced depression scores in older adults may improve certain common symptoms among these variables, such as lack of energy, motivation, eating disorders and sleep, achieving an improvement in their level of physical activity^24^. The negative impact of depression on the physical, social and health cost aspects of older adults suggests the importance of early interventions and strategies aimed at their identification and treatment. Thus, holding therapeutic groups for screening of susceptible older adults, observation of the presented symptoms, help in coping with psychological issues and a multidisciplinary approach are essential.

This study has limitations such as the self-reporting of morbidities, since older adults could forget or not adequately remember/be aware of their morbidities. However, by understanding the changes in frailty conditions among older adults after hospital discharge and identifying its predictor variables, we will have more subsidies for planning and implementing early interventions. Moreover, these results may help future research which may contribute to the improving care assistance aiming at prevention, improvement or staging of this syndrome.

## Conclusion

The highest percentages of changes in frailty condition occurred from non-frail to pre-frail older adults and from frail to pre-frail. No changes in the frailty condition from non-frail to frail older adults have been verified. Death occurred in frail and pre-frail older adults. The variables predicting changes in frailty conditions were statistically significant only for the components of the frailty phenotype. The increase in number of morbidities was more likely to worsen self-reported exhaustion and/or fatigue. In the improving group, the increase in IADL dependence constituted a greater chance of unintentionally losing weight, and a decrease in the indication of depression scores was considered a protection factor for low physical activity level.
